# InhA, the enoyl-thioester reductase from *Mycobacterium tuberculosis* forms a covalent adduct during catalysis

**DOI:** 10.1074/jbc.RA118.005405

**Published:** 2018-09-14

**Authors:** Bastian Vögeli, Raoul G. Rosenthal, Gabriele M. M. Stoffel, Tristan Wagner, Patrick Kiefer, Niña Socorro Cortina, Seigo Shima, Tobias J. Erb

**Affiliations:** From the Departments of ‡ Biochemistry and Synthetic Metabolism and; § Microbial Protein Structure, Max-Planck-Institute for Terrestrial Microbiology, 35043 Marburg, Germany and; the ¶ Institute of Microbiology, ETH Zürich, 8093 Zürich, Switzerland

**Keywords:** enzyme catalysis, enzyme mechanism, reductase, enzyme structure, enzyme kinetics, enoyl-CoA reductase, InhA, Mycobacterium tuberculosis, pericyclic reaction, short-chain dehydrogenase/reductase

## Abstract

The enoyl-thioester reductase InhA catalyzes an essential step in fatty acid biosynthesis of *Mycobacterium tuberculosis* and is a key target of antituberculosis drugs to combat multidrug-resistant *M. tuberculosis* strains. This has prompted intense interest in the mechanism and intermediates of the InhA reaction. Here, using enzyme mutagenesis, NMR, stopped-flow spectroscopy, and LC–MS, we found that the NADH cofactor and the CoA thioester substrate form a covalent adduct during the InhA catalytic cycle. We used the isolated adduct as a molecular probe to directly access the second half-reaction of the catalytic cycle of InhA (*i.e.* the proton transfer), independently of the first half-reaction (*i.e.* the initial hydride transfer) and to assign functions to two conserved active-site residues, Tyr-158 and Thr-196. We found that Tyr-158 is required for the stereospecificity of protonation and that Thr-196 is partially involved in hydride transfer and protonation. The natural tendency of InhA to form a covalent C2-ene adduct calls for a careful reconsideration of the enzyme's reaction mechanism. It also provides the basis for the development of effective tools to study, manipulate, and inhibit the catalytic cycle of InhA and related enzymes of the short-chain dehydrogenase/reductase (SDR) superfamily. In summary, our work has uncovered the formation of a covalent adduct during the InhA catalytic cycle and identified critical residues required for catalysis, providing further insights into the InhA reaction mechanism important for the development of antituberculosis drugs.

## Introduction

Tuberculosis remains one of the deadliest infectious diseases (1). With increasing multidrug-resistant strains of *Mycobacterium tuberculosis*, the cause of the disease, the need for next generation treatments increases (1). The enoyl-acyl carrier protein (enoyl-ACP)^3^ reductase InhA is a major target for the clinically relevant antibiotics isoniazid and ethionamide (2). In the light of emerging multidrug-resistant *M. tuberculosis* strains, InhA remains a prime candidate for drug design (3). Therefore, the recent decade has seen intensive research focusing on developing new inhibitors against this enzyme (4).

**Figure 1.**
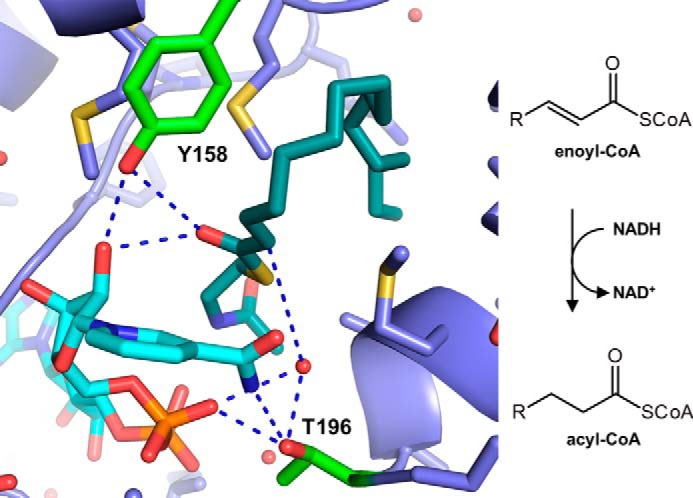
**Active-site architecture of InhA in tripartite structure containing NAD^+^ (*cyan*) and C-16 acyl-substrate analogue (*dark cyan*) (PDB code 1BVR (3)).** Tyrosine 158 (*green*) is positioned for hydrogen bonding with the carbonyl of the acyl-thioester and the hydroxyl group of the NAD^+^ ribose. Threonine 196 (*green*) is positioned below the carboxamide of NAD^+^ and within hydrogen bond distance to the β-phosphate and a water molecule, which is positioned below the Cα of the substrate

InhA catalyzes the NADH-dependent reduction of enoyl-ACP in the biosynthesis of fatty and mycolic acids, which form an essential component of the membrane and cell wall of *M. tuberculosis*, respectively (5, 6). The reaction mechanism is postulated to start with a direct hydride transfer from the C4 of NADH to the β-carbon of the enoyl-ACP, followed by formation of an enolate anion, which is subsequently protonated stereospecifically to the pro-(2*R*) position of the α-carbon (3, 7). The source of the proton, however, remains unknown and was suggested to originate from a solvent water because of the lack of any protic amino acid residues positioned close enough to serve as proton donor (7). Tyrosine 158, one of the only protic groups in the active site of InhA, was shown by mutagenesis studies to be involved in catalysis. When mutated to phenylalanine (Y158F), the enzyme is reported to lose ∼1 order of magnitude in catalytic efficiency. The hydroxyl group of tyrosine 158 was proposed to provide electrophilic stabilization of the transition state(s) by hydrogen bonding to the carbonyl of the substrate (Fig. 1) (8). However, the Y158F variant did not show a significantly different solvent kinetic isotope effect (^D_2_O^V) compared with the WT enzyme (WT: ^D_2_O^V = 1.5 ± 0.2; Y158F ^D_2_O^V = 1.4 ± 0.2) (7).

Here we show that the Y158F variant forms and accumulates a previously overlooked covalent C2-ene adduct between the NADH cofactor and the CoA thioester substrate during catalysis. We use the isolated adduct as “molecular probe” to demonstrate that Tyr-158 is not deficient in hydride transfer but directly affected in the protonation step. Using stopped-flow spectroscopy and HPLC–MS, we show that InhA WT forms the same intermediate also in its native catalytic cycle. The detection of the C2-ene adduct in the catalytic cycle of InhA calls for a careful reconsideration of the reaction mechanism of InhA and related NAD(P)H-dependent oxidoreductases. In addition, we describe novel tools to study the catalytic cycle of InhA and potential new avenues to eventually inhibit this important drug target.

## Results

### Tyrosine 158 and threonine 196 are involved in catalysis

Analysis of a crystal structure containing the cofactor NAD^+^ and a 16C-acyl-SNAC substrate analogue suggested a water molecule bound by threonine 196 that could be involved in catalysis and particularly in proton donation (Fig. 1) (3). We therefore characterized active site variants of Thr-196 as well as the previously suggested variants of Tyr-158 using octenoyl-CoA as a substrate for the reaction (Table 1 and Fig. S1). The Y158F variant retained only 1.8% of the WT *k*_cat_ with a 2-fold increase of the *K_m_* for octenoyl-CoA, which corresponds well with previous studies (7). For an Y158S mutant, contrary to a previous report (7), we saw an even stronger decrease in *k*_cat_ to only 0.16% of WT activity. The Thr-196 variants, on the other hand, also showed a strong effect on catalysis. T196A retained 1.0%, and the larger, isosteric T196V variant retained 0.15% of WT *k*_cat_. The *K_m_* for octenoyl-CoA was increased by more than 5-fold in both mutants, whereas the *K_m_* for NADH did not change significantly compared with WT. In summary, our kinetic characterization showed that both Tyr-158 and Thr-196 are involved in catalysis.

**Table 1:** **Kinetic parameters of InhA WT and variants** For the assays of the kinetics with octenoyl-CoA, NADH was kept constant at 300 μm, for the ones with NADH octenoyl-CoA was kept at 4 mm. All assays were measured in 30 mm PIPES, 150 mm NaCl, pH 6.8, at 30 °C at 340 nm. C2 adduct was added in powder form directly from liquid nitrogen, and its consumption was measured at 385 nm. Michaelis–Menten curves are shown in Fig. S1. NA, not applicable; enzyme catalyzes backwards reaction to substrate starting from C2-ene adduct.

Enzyme variant	Substrate	*k*_cat,app_	*K_m_*_,app_
		*s*^−*1*^	*mm*
InhA WT	Octenoyl-CoA	3.6 ± 0.2	0.8 ± 0.1
	NADH	3.5 ± 0.2	0.09 ± 0.01
	C2-ene adduct	3.0 ± 0.2	0.011 ± 0.002
InhA Y158F	Octenoyl-CoA	0.088 ± 0.005*^a^*	2.0 ± 0.3
	NADH	0.079 ± 0.001*^a^*	0.01 ± 0.001
	C2-ene adduct	NA	NA
InhA Y158S	Octenoyl-CoA	0.0055 ± 0.0002	0.9 ± 0.1
	NADH	0.0059 ± 0.0003	0.056 ± 0.009
	C2-ene adduct	0.23 ± 0.02	0.030 ± 0.006
InhA T196A	Octenoyl-CoA	0.050 ± 0.003	7.6 ± 0.7
	NADH	0.012 ± 0.001*^b^*	0.059 ± 0.011
	C2-ene adduct	0.077 ± 0.008	0.01 ± 0.003
InhA T196V	Octenoyl-CoA	0.0073 ± 0.0008	4.3 ± 1.0
	NADH	0.0051 ± 0.0003*^b^*	0.11 ± 0.02
	C2-ene adduct	0.056 ± 0.003	0.022 ± 0.003

*^a^* Kinetic parameters for C2-ene adduct production. Enzyme does not catalyze full reaction.

*^b^* NADH kinetics for T196A and T196V were not measured at octenoyl-CoA saturation because of solubility constrains leading to a lowered *k*_cat,app_ for these assays.

### Tyrosine 158 is essential for stereospecificity of protonation

To assess the function of Tyr-158 and Thr-196 in more detail, we next used isotopic labeling to determine the protonation stereochemistry of InhA WT and its variants. We performed enzyme reactions in deuterated buffer and used a stereospecific oxidase Acx4 from *Arabidopsis thaliana* to quantify the incorporation pattern of deuterium at the 2 position (Fig. S2). InhA WT incorporated the deuteron with an efficiency of 99 ± 1% specifically into the *2R* position, which is in line with previous data (9). The Y158F variant incorporated deuterium with an efficiency of 57 ± 2% into the *2R* position, which indicated that this variant lost stereospecificity of proton donation. Surprisingly the Y158S variant only partially lost stereospecificity of proton donation. This might be due to a water molecule in the active site able to compensate for the loss of the hydroxyl group of Tyr-158 as previously suggested by Parikh *et al.* (7). Both variants T196A and T196V showed only a partial loss in stereospecificity, incorporating the deuteron into the *2R* position with 91 ± 1 and 77 ± 1%, respectively (Table 2). These results suggested that Tyr-158 contributes stronger to the stereospecificity of proton donation than Thr-196. We next aimed at determining the kinetic isotopic effects (KIEs) of protonation in the Y158F variant.

**Table 2:** **Stereospecificity of protonation in InhA WT and variants as determined by the label incorporation in the 2S position starting with substrates or with C2-ene adduct** A 200-μl assay contained 400 μm NADH and 300 μm octenoyl-CoA in deuterated 30 mm PIPES, 150 mm NaCl buffer, pD 6.8, and were started with 12.5 μm InhA WT, 22.6 μm InhA Y158F, 70.3 μm Y158S, 23.9 μm InhA T196V, and 40 μm InhA T196A. The reactions were followed spectrophotometrically at 360 nm and run at 30 °C until complete consumption of NADH (∼1 min for WT and 3 h for Y158F, Y158S, and T196A) except for the assay containing T196V, which was stopped after 7 h after approximately 50% of NADH was consumed. Detailed workup of the assay and analysis is described under “Experimental procedures.”

Enzyme variant	No label (2R)	+1 label (2S)
	%	%
InhA WT	99 ± 1	1 ± 1
InhA Y158F	57 ± 2	43 ± 2
InhA Y158S	79 ± 1	21 ± 1
InhA T196A	91 ± 1	9 ± 1
InhA T196V	77 ± 1	23 ± 1

### KIE measurements confirm that tyrosine 158 is directly involved in protonation

Parikh *et al.* (7) reported almost identical KIEs on protonation for InhA WT and Y158F (WT: ^D_2_O^V = 1.51; YF: ^D_2_O^V = 1.39). These KIEs were measured indirectly by following the consumption of the cosubstrate NADH in H_2_O or D_2_O, assuming that reduction and protonation are coupled. NADH consumption, however, does not necessarily remain coupled to protonation in the different enzyme variants, so that the intramolecular KIE on protonation might become masked when indirect measurements are used (10). Therefore, we decided to apply an alternative method that would allow us to directly measure the intramolecular KIE on protonation (^D^*k*_obs_) (11–13). Here, the intramolecular ^D^*k*_obs_ on protonation is determined by running assays in buffers with different H_2_O and D_2_O contents and fitting the isotopic composition of the products to Equation 1 (12).

(Eq. 1) productHproductD= Dkobs·fH2OfD2O

Using this direct method, we measured a ^D^*k*_obs_ of 1.74 ± 0.06 for InhA WT with hexenoyl-CoA as substrate. The ^D^*k*_obs_ decreased with increasing chain length of the substrate to 1.14 ± 0.01 for dodecenoyl-CoA (Table 3), indicating an increasing commitment factor with increasing chain length. Compared with WT InhA, the Y158F variant showed a significantly increased ^D^*k*_obs_ (Table 3). The ^D^*k*_obs_ decreased with substrate length from 3.9 ± 0.3 with hexenoyl-CoA to 2.4 ± 0.1 with dodecenoyl-CoA. The strong difference in ^D^*k*_obs_ between WT and Y158F indicates that the Y158F variant is affected in the protonation step. At the same time, the apparent differences observed in KIEs between the NADH consumption and the deuterium incorporation method suggests that the reduction and protonation steps become (partially) uncoupled in the Y158F variant.

**Table 3:** **^D^*k*_obs_ of InhA WT and Y158F** The ^D^*k*_obs_ was measured by quantifying the discrimination between H and D addition at enoyl-CoA concentrations that were 20-fold below *K_m_* to keep the commitment factor low and a NADH concentration of 10 mm, more than 100-fold over *K_m_*, to avoid a change in the commitment factor because of a change in NADH concentration.

Substrate	InhA WT	InhA Y158F
Hexenoyl-CoA	1.74 ± 0.14	3.9 ± 0.3
Octenoyl-CoA	1.28 ± 0.04	3.4 ± 0.1
Dodecenoyl-CoA	1.14 ± 0.03	2.4 ± 0.1

### Y158F variant accumulates a covalent adduct between crotonyl-CoA and NADH

**Figure 2.**
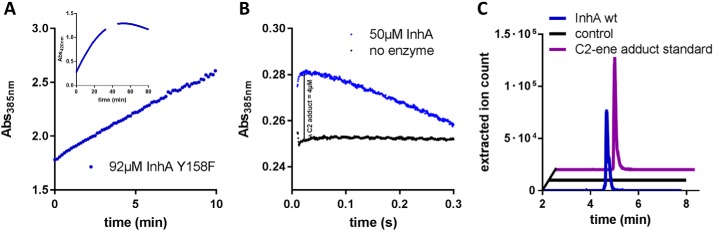
**Detection of C2-ene adduct in InhA Y158F and WT.** *A*, production of C2-ene adduct by InhA Y158F. The assay contained 15.4 mm octenoyl-CoA, 23.6 mm NADH, and 92 μm InhA Y158F. C2-ene adduct production was followed at 385 and 420 nm (*inset*). The assay was quenched when C2-ene formation plateaued after 32 min and directly injected into the HPLC for purification and further characterization. *B*, stopped flow analysis of InhA WT at 385 nm; syringe 1 contained 100 μm InhA WT (*blue line*), syringe 2 contained 4 mm octenoyl-CoA and 1 mm NADH all in 30 mm PIPES, pH 6.8, 150 mm NaCl buffer. In the control syringe 1 contained only buffer without enzyme (*black line*). The data shown are the averages of triplicates for each condition. *C*, LC–MS analysis of InhA. The assay was directly injected after 60-s incubation at room temperature during steady-state catalysis and contained 50 μm InhA WT, 250 mm NADH, and 50 mm octenoyl-CoA. In a control experiment containing 250 mm NAD^+^ instead of NADH, no C2-ene adduct was detected.

To investigate the uncoupling of the reduction and protonation steps in the Y158F variant, we characterized the enzyme's reaction in more detail. Spectrophotometric analysis of the Y158F reaction showed a transient increase of absorbance with a maximum at 375 nm, reminiscent of the formation of transient reaction intermediates that were observed recently in the enoyl-reductase Etr1p and other enzymes from the medium chain dehydrogenase/reductase superfamily (Fig. 2*A*) (14–16). This observation indicated that the reaction of the Y158F variant proceeded stepwise via accumulation of an intermediate species. We next isolated the intermediate species via preparative HPLC and characterized it further by UV-visible spectroscopy (Fig. S3*A*) and high-resolution MS.

The isolated species was labile (see below) and had a mass-to-charge ratio (*m*/*z*) of 779.1717, which matched the expected mass of a double charged direct adduct between NADH and octenoyl-CoA (Fig. S4). Two-dimensional (COSY) NMR confirmed CoA and NADH substructures in the intermediate and identified a covalent bond between the C2 proton of the NADH nicotinamide ring and the α-proton of the octenoyl-CoA (Fig. S5). In summary, these experiments showed that a covalent C2-ene adduct accumulates in solution in the Y158F variant during catalysis. However, in contrast to similar C2-ene adducts previously reported for enzymes of the medium-chain dehydrogenase/reductase superfamily (14–16), the adduct from InhA Y158F is proposed to feature the opposite stereochemistry at the α-carbon (*i.e.* (2*S*)-C2), because of the binding geometry of the NADH cofactor and the substrate at the active site. It is the first report of an enzyme from the short chain dehydrogenase/reductase superfamily, suggesting that these adducts are a more common feature of nicotinamide-dependent chemistry.

The isolated (2*S*)-C2-ene adduct decayed uncatalyzed in solution into the products octanoyl-CoA and NAD^+^ with a decay constant of 0.186 ± 0.001 min^−1^ (Fig. S3*B*). This uncatalyzed decay into the final reaction products provides an explanation for the only partial loss of activity observed upon mutation of Y158F. Although the Y158F variant is affected in the protonation step, the enzyme can still form the (2*S*)-C2-ene adduct, which then decomposes into the final products in solution. The spontaneous decay of the intermediate in solution explains the lack of stereospecificity of deuterium incorporation into octanoyl-CoA produced by Y158F. To rule out that the Y158F mutation caused major changes to the active site, we cocrystallized Y158F with NAD^+^. Analysis of the crystal structure confirmed that the active site geometry was not perturbed compared with WT (Fig. S6, PDB code 6EP8, Table S1)

### The C2-ene adduct is also formed in the WT enzyme

The detection of the (2*S*)-C2-ene adduct in Y158F prompted the question of whether this covalent adduct is only formed in this variant or whether the (2*S*)-C2-ene adduct is a true intermediate in the catalytic cycle of the WT enzyme. Using stopped-flow spectroscopy on the WT, we observed an increase of absorption at 385 nm, the wavelength at which the extinction coefficients of NADH and the (2*S*)-C2-ene adduct differ most. The increase appeared in the first turnover within the first 50 ms and corresponded to ∼4 μm (2*S*)-C2-ene adduct, which in turn corresponded to 8% of the total active sites of enzyme in the assay (Fig. 2*B*). This result suggested that the (2*S*)-C2-ene adduct is formed in the catalytic cycle of the WT enzyme. Our finding was confirmed by directly injecting an enzyme assay with WT InhA during steady-state catalysis into an HPLC–ESI–MS, which demonstrated existence of the (2*S*)-C2-ene adduct also under steady-state conditions (Fig. 2*C*). In summary, these results suggest that the (2*S*)-C2-ene adduct is formed during the catalytic cycle of InhA WT and not simply as an artifact of the Y158F mutation.

### C2-ene adduct as a “molecular probe” confirms the role of tyrosine 158 in protonation

The (2*S*)-C2-ene adduct represents an intermediary state of catalysis; in the covalent adduct the reduction has already taken place, whereas the protonation has not. This specific feature allows to use the (*2S*)-C2-ene adduct as a “molecular probe” to test protonation independent of the (preceding) reduction step (14). Indeed, purified (*2S*)-C2-ene adduct was catalytically competent and served as *bona fide* substrate for InhA WT at a *k*_cat_ of 3.5 ± 0.2 s^−1^ and at a *K_m_* of 11 ± 2 μm (Table 1).

We next used (*2S*)-C2-ene adduct to specifically probe the protonation step of InhA WT and different variants. WT converted the adduct to the products octanoyl-CoA and NAD^+^ as judged from the uniform decrease of the adduct peak in the UV-visible spectrum (Fig. S7). In contrast, in the Y158F variant, the peak maximum shifted from 385 to 340 nm, which indicated formation of NADH and thus partial resolution of the (2*S*)-C2-ene adduct back into the two substrates. From the absorbance increase, we estimated that most of the (2*S*)-C2-ene adduct was converted back to octenoyl-CoA and NADH. Thus, the Y158F variant catalyzed mainly the back reaction. This result is in line with the fact that the protonation step—and thus product formation from the (2*S*)-C2-ene adduct—is impaired by the Y158F mutation (Fig. S7). The Y158S variant on the other hand was still able to convert the (2S)-C2-ene adduct into the product. Compared with the overall reaction, in which the *k*_cat_ was reduced to 0.16% of WT activity (0.0059 ± 0.0003 s^−1^; Table 1), the *k*_cat_ for the (2S)-C2-ene adduct was 40-fold increased to 7.8% of WT activity (0.23 ± 0.02 s^−1^; 7.8% of the WT), indicating that the protonation is less affected in this variant than hydride transfer. It was previously suggested that a water molecule bound to the serine can serve as the resolving electrophile (7). Our data are well in line with this hypothesis and shows that Tyr-158 is involved in hydride transfer, most likely by stabilizing the oxyanion, and protonation.

The T196V and T196A variants were also able to convert the (2*S*)-C2-ene adduct further into the products, indicating that these variants are less affected in catalyzing the protonation step (Fig. S7). Note, that both Thr-196 variants showed a larger decrease in *k*_cat_ for the overall reaction (T196V showed 0.15% WT activity) compared with the reaction starting with the (2*S*)-C2-ene adduct (T196V showed 1.9% WT activity). This indicates that Thr-196 is partially involved in hydride transfer and protonation but contributes less to the protonation reaction than Tyr-158 (Table 1).

## Discussion

Understanding the mechanism of enzymes is a prerequisite to be able to manipulate their function (17). Here we provide new insights into the catalytic cycle of the enoyl-CoA reductase InhA, the major drug target of *M. tuberculosis*. Our data show that InhA forms a covalent adduct between the nicotinamide cofactor and the enoyl-CoA substrate during catalysis as observed in both pre–steady-state (stopped-flow spectroscopy) as well as steady-state (LC–MS) measurements. Intriguingly, similar dihydropyridine-substrate adducts were observed recently in chemical model reactions (18), the biosynthesis of the natural product sanguinarine (19), and the catalytic cycle of enoyl-CoA reductases of the medium-chain dehydrogenase (MDR) superfamily (15, 16). The detection of the (2*S*)-C2-ene adduct in InhA, an enzyme from the SDR superfamily, complements these recent observations. The SDR and MDR superfamilies both represent very ancient protein folds that emerged and diversified very early from the communal pool during cellular evolution (20). MDRs and SDRs share the cofactor binding Rossman fold but have a different overall molecular architecture and different catalytic mechanisms (20). They can be found in all domains of life. There are 82 SDR and 25 MDR genes in humans alone that catalyze a wide range of reactions, covering half of all enzyme activity types (tree of six EC classes) (20). The observation of C2-ene adducts in both ancient nicotinamide-dependent enzyme superfamilies suggests that the formation of such covalent adducts is not a singularity of one enzyme or one enzyme superfamily but eventually a more general catalytic principle at the active site of nicotinamide-dependent oxidoreductases and of the nicotinamide cofactors themselves.

**Figure 3.**
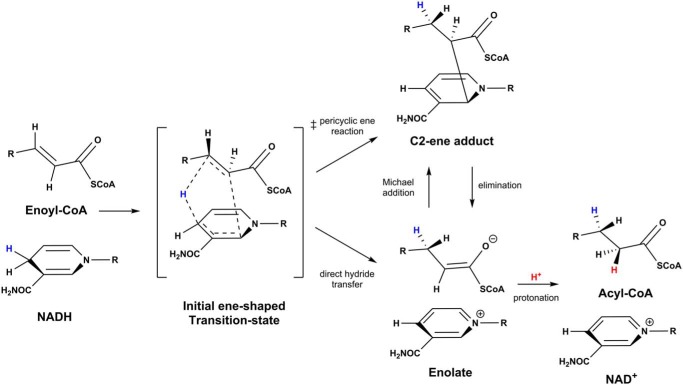
**Proposed reaction pathways for InhA and related oxidoreductases.** In a first step, the substrates enoyl-CoA and NADH form an ene-shaped transition state. The observed C2-ene adduct can then be formed either directly through a pericyclic ene reaction or via a enolate formed from direct hydride transfer through a Michael-addition reaction. The C2-ene adduct can then be resolved through an elimination reaction, and the enolate abstracts a proton to form the reduced acyl-CoA product.

How is the covalent adduct formed and why? Based on their studies on model compounds, Libby and Mehl (18) suggested the alignment of the reactants in an ene-like transition state (Fig. 3). The character and position of the reactants, as well as the electronic environment (*e.g.* the active site of the catalysts), will strongly influence the way the hydride is transferred. One (extreme) possibility is that hydride transfer proceeds in a pericyclic fashion, resulting in the direct formation of the (2*S*)-C2-ene adduct. The (2*S*)-C2-ene adduct would then be resolved via the enolate to the final products in subsequent steps. Alternatively, the hydride is transferred to the Cβ of the enoyl-CoA ester according to the “classical” mechanism directly forming the enolate. The enolate can either react further to the reaction product (*e.g.* by protonation) or reattack the nicotinamide cofactor in a Michael-type addition to form the (2*S*)-C2-ene adduct (Fig. 3). We note that the lack of good electrophiles in proximity to the Cα of the enolate favors such a Michael addition, as for instance is the case in enoyl-CoA carboxylase/reductases when CO_2_ is omitted (16) or Etr1p when the proton donor is mutated (14). In available crystal structures of InhA with cofactor and product, no obvious proton donor is positioned close to the Cα (3), indicating that the formation and accumulation of the (2*S*)-C2-ene adduct might be a logical consequence of the active-site geometry of InhA.

We note that formation of the (2*S*)-C2-ene adduct also provides an explanation for the puzzling stereochemistry of protonation in InhA, which proceeds from the same side as the hydride is transferred, although an obvious proton donor is absent from this side (8). (2*S*)-C2-ene adduct formation leads to a change in hybridization of the Cα of enoyl-CoA from sp^2^ to sp^3^, which would bring the Cα closer to the phenolic hydroxyl group of Tyr-158 (Fig. S8). This repositioning of the substrate would put Tyr-158 into the position for a proton transfer after the (2S)-C2-ene adduct is decomposed back into the enolate and NAD^+^ (Fig. S8). This mechanism could explain the observed involvement of Tyr-158 in both the hydride transfer, as well as the proton transfer.

In addition to mechanistic implications, the discovery of a covalent adduct in the catalytic cycle of InhA provides the basis to prepare new tools to interrogate and eventually interfere with the mechanism of InhA. Using the (2*S*)-C2-ene adduct as molecular probe allowed us to assess the function of individual amino acid residues at the active site of InhA and quantify their contribution to either step of the reaction. At the same time, the (2S)-C2-ene adduct could inspire the design of a new class of mechanistic inhibitors of InhA and related NAD(P)H-dependent oxidoreductases. Note that compounds that are able to trap substrates at the covalent intermediate stage are expected to be high-affinity binders of these enzymes. Such compounds could act in analogy to finasteride, a mechanism-based inhibitor of 5α-reductase that forms a similar covalent adduct between the C4 of NADH and the prodrug within the active site of the enzyme (21). Thus, exploiting the natural tendency of InhA to form covalent adducts with NADH might prove to be a convenient way to inhibit this key target of *M. tuberculosis*.

## Experimental procedures

### Chemicals

Hexenoic- and octenoic-acid (synthesis grade) were purchased from Sigma–Aldrich AG, CoA, and DNaseI from Roche Diagnostics; Na_2_NADH (98%) was purchased from Carl Roth GmbH. All salts and solvents were of analytical grade or better. Dodecenoic acid was synthesized from decanal via Knoevenagel condensation with malonic acid according to a previously described protocol (22). Enoyl-CoAs were synthesized using the mixed anhydride method adapted from a previously described protocol (22, 23). The unsaturated acid (156 μmol) and triethylamine (156 μmol) were dissolved in CH_2_Cl_2_ (3 ml) and stirred at 23 °C for 30 min. The reaction was cooled to 4 °C, and ethylchloroformate (156 μmol) was added. After 2 h the solvent was evaporated, the crude product was dissolved in *N*,*N*-dimethylformamide (4.5 ml) and added to a stirring solution of CoA-trilithium salt (76 μmol) in 0.4 m KHCO_3_ (4.5 ml). The reaction procedure was monitored by mixing 5 μl of reaction mixture with 35 μl of an aqueous 5,5′-dithiobis-2-nitrobenzoic acid (Ellman's reagent) solution. Upon completion the reaction was acidified to pH 3–4 with formic acid, diluted to 50 ml with H_2_O, and lyophilized. The product was resuspended in H_2_O and purified by reverse-phase HPLC over a Gemini 10-μm NX-C18 110 Å, 100 × 21.2-mm, AXIA packed column (Phenomenex) using a gradient from 5 to 65% (hexenoyl-CoA) and to 95% (octenoyl-, dodecenoyl-CoA) over 15 min with 25 mm ammonium formate, pH 8.1, as the aqueous phase. Fractions containing the product were pooled, lyophilized, and stored at −20 °C.

### Cloning and mutagenesis

The InhA WT gene from *M. tuberculosis* present in plasmid pET15b (gift from Prof. John Blanchard, Albert Einstein College of Medicine, New York, NY) was used as received to express InhA WT. InhA variants were generated with the QuikChange® site-directed mutagenesis kit (Stratagene, La Jolla, CA) using 60 ng of template plasmid and the following primer pairs: Y158F, CGATGCCGGCCTTCAACTGGATGAC and GTCATCCAGTTGAAGGCCGGCATCG; Y158S, CGATGCCGGCCTCCAACTGGATGAC and GTCATCCAGTTGGAGGCCGGCATCG; T196A, CAGGCCCTATCCGGGCGCTGGCGATGAGTG and CACTCATCGCCAGCGCCCGGATAGGGCCTG; and T196V, GGCCCTATCCGGGTGCTGGCGATGAG and CTCATCGCCAGCACCCGGATAGGGCC.

### Protein expression and purification

The His-tagged proteins were expressed in *Escherichia coli* BL21 (DE3) or *E. coli* BL21-AI^TM^ (DE3) in terrific broth by inducing at *A*_600_ = 1.5 with 0.5 mm isopropyl-β-d-thiogalactoside and 2.5 mm arabinose when using *E. coli* BL21-AI^TM^ (DE3) at 25 °C for 12–16 h. Harvested cells were lysed in 2.5 ml of lysis buffer per gram cells: lysis buffer contained 500 mm NaCl, 20 mm Tris-HCl, 10% (v/v) glycerol, pH 7.9, with 1 mm MgCl_2_ and DNaseI at 1 μg/ml. The cells were lysed by sonication and subsequently centrifuged at 50,000 × *g* for at least 30 min at 4 °C. The clear supernatant was applied to a 1-ml HisTrap FF column and washed with 24% buffer B for 30–40 column volumes, until the UV 280-nm absorbance did not decrease anymore. The protein was eluted with 100% buffer B. Buffer A contained 500 mm NaCl, 20 mm Tris-HCl, pH 7.9, and Buffer B was identical to buffer A with the addition of 250 mm imidazole. The eluted protein was desalted into 30 mm PIPES, 150 mm NaCl, pH 6.8, within 30 min from eluting, because previous reports mention that the protein can precipitate in the elution buffer (7). The enzyme was kept in this buffer at 4 °C until it was used.

### Determining the stereochemistry of protonation

Isotopic label incorporation experiments were done analogous to a previously described method (see reaction scheme in Fig. S2) (14). The protein storage buffer of InhA WT and variants was exchanged using three subsequent ultracentrifugation steps (Amicon Ultra 0.5-ml centrifugal filters; Merck Millipore) diluting the original buffer over 10,000× with deuterated 30 mm PIPES, 150 mm NaCl buffer, pD 6.8. A 200-μl assay contained 400 μm NADH and 300 μm octenoyl-CoA in deuterated 30 mm PIPES, 150 mm NaCl buffer, pD 6.8 and was started with 12.5 μm InhA WT, 22.6 μm InhA Y158F, 70.3 μm Y158S, 23.9 μm InhA T196V, and 40 μm InhA T196A. The reactions were followed spectrophotometrically at 360 nm and run at 30 °C until complete consumption of NADH (∼1 min for WT and 3 h for Y158F, Y158S, and T196A) except for the assay containing T196V, which was stopped after 7 h after ∼50% of NADH was consumed. 20 μl of 50% formic acid was added to quench the reaction. The octanoyl-CoA was purified by HPLC, lyophilized, and resuspended in 100 mm Tris-HCl, pH 8.0. Label incorporation was checked by HPLC–ESI–MS. 7.9 μm Acx4 was added to the samples, and the reaction mixtures were incubated for 10 min at 30 °C, quenched by adding 5% formic acid, and analyzed by HPLC–ESI–MS (Table 2). To make sure that the oxidase Acx4 does not unspecifically scramble the signal in the α position, an assay containing 300 μm octanoyl-CoA, 0.13 μm Acx4 was run in deuterated 100 mm Tris-DCL, pD 8.0, quenching the assay at different time points by adding 5% formic acid. Over the time course of 90 min, no incorporation of a deuterium signal in either octanoyl-CoA or octenoyl-CoA could be detected.

### KIE measurements

Reactions for the measurement of the KIEs in InhA WT and Y158F contained 30 mm PIPES buffer, pH 6.8, 150 mm NaCl, 10 mm NADH, 260 μm hexenoyl-CoA, 26 μm octenoyl-CoA, or 4.6 μm dodecenoyl-CoA. H_2_O and D_2_O were added according to the desired percentage of D_2_O: between 10 and 70% (v/v). The enzymes were equilibrated with the partially deuterated buffer for at least 2 h before the assay was started with the addition of the enoyl-CoA substrate (enoyl-CoA substrates were dissolved as 25-fold stock solutions in 50% DMSO). Hexenoyl-CoA and octenoyl-CoA assays contained 147 nm of InhA WT or InhA Y158F, and the dodecanoyl-CoA assays contained 29 nm of either enzyme. Assays with InhA WT were quenched by the addition of 5% formic acid after 4 min for octenoyl-CoA and dodecanoyl-CoA and after 20 min for hexanoyl-CoA. Assays with InhA Y158F were quenched after 150 min for all three substrates.

### C2-ene adduct purification and characterization

A mixture containing 15.5 mm octenoyl-CoA, 23.7 mm NADH, and 92.5 μm InhA Y158F in 200 mm Tris-HCl (pH 7.4 ml) was reacted at 4 °C until the absorption at 400 nm reached a maximum. The reaction was purified on an Agilent HPLC system using a flow rate of 25 ml min^−1^ and using a Gemini 10-μm NX-C18 110 Å, 100 × 21.2-mm, AXIA packed column (Phenomenex). The elution of the adduct was followed at 370 nm and collected directly into liquid nitrogen. Upon lyophilization (0.01 mbar, −55 °C), the compound appeared as an intense yellow powder that was stored at −80 °C. For spectrophotometric analysis C2-ene adduct was dissolved in 30 mm PIPES buffer, pH 6.8, 150 mm NaCl. The absorption spectrum of the C2-ene adduct (Fig. S3*A*) and the uncatalyzed decay of C2-ene adduct at 30 °C (Fig. S3*B*) was determined on a Cary-60 UV-visible spectrometer (Agilent) using quartz cuvettes (1-, 3-, or 10-mm diameter; Hellma). For NMR spectroscopy, the C2-ene adduct was dissolved in D_2_O 25 mm Na_2_DPO_4_, pH 7.9, and measured at 4 °C at 600 MHz (Fig. S5).

### Stopped-flow spectroscopy

Measurements were recorded on a thermostatted stopped flow unit (SFM-20 connected to a MOS-200, equipped with a Xe(Hg) lamp and a TC-100/10 cuvette; Bio-Logic Science Instruments SAS, Claix, France) set to 30 °C. Syringe 1 contained 1 mm NADH and 4 mm octenoyl-CoA in 30 mm PIPES, 150 mm NaCl buffer, pH 6.8, and syringe 2 contained 100 μm of InhA WT in the same buffer. For the controls either syringe 1 or 2 contained only buffer. The data were collected at 385 nm every 0.5 ms for the first 4 s and then every 0.5 s up to a total of 90 s. Each assay and control was repeated at least four times, and the resulting traces were averaged to reduce noise.

### Protein crystallization

For protein crystallization, the HisTrap-purified InhA was incubated with thrombin to cleave off the His tag (120 units thrombin/8 mg InhA) for 2 h at room temperature. The solution was then passed through a HisTrap column equilibrated in 30 mm PIPES, 150 mm NaCl, pH 6.8. The flow-through was injected onto a Ge HiLoad 16/600 Superdex 200-pg column running on buffer containing 30 mm PIPES, 150 mm NaCl, pH 6.8. Crystallization of polyhistidine tag cleaved InhA from *M. tuberculosis* was performed under air at 14–20 °C with a protein concentration of 10 mg/ml. For all crystallization experiment, 3–10 mm of NADH was added to the protein solution, and the protein sample was filtered through a 0.2-μm filter. The 24-well crystal plate (Combiclover Junior; Jena Bioscience) was used for sitting drop, and the crystallization conditions from Chollet *et al.* (24) was reproduced. The best crystals appeared in drops of 2 μl of enzyme solution, 1 μl of the reservoir solution, and 1 μl of distilled water. The reservoir solution contained 100 mm HEPES/NaOH, pH 7.5, 50 mm sodium citrate, pH 6.5, 7–9% 2-methyl-2,4-pentanediol. Bipyramidal-shaped crystals appeared typically within 2–3 weeks in this condition.

### Structural analysis

The crystals were cryoprotected by soaking with 25% glycerol (v/v) in the crystallization solution for 3–5 s prior freezing in liquid nitrogen. The diffraction experiments were performed at 100 K on Beamline BM30A (French Beamline for Investigation of Proteins (FIP)) at the European Synchrotron Radiation Facility (Grenoble, France) equipped with a CCD detector (ADSC Q315r). The data were processed with XDS (25) and scaled with SCALA from the ccp4 suite (26). The structure was determined using the InhA WT from *M. tuberculosis* inhibited with the active metabolite of isoniazid (PDB code 4TRO) as a template for MOLREP. The model was manually constructed with COOT (27) and refined by PHENIX (28) including hydrogens. The final model was validated by using the MolProbity server (http://molprobity.biochem.duke.edu)^4^ (29) and deposited without hydrogens. Data collection and refinement statistics of the model are listed in Table S1. The figures were generated and rendered with PyMOL (version 1.5; Schrödinger, LLC) for a comparison of the solved structure of InhA Y158F (PDB code 6EP8) with the previously solved WT structure (PDB code 1BVR (3); Fig. S6).

### Spectrophotometric enzyme assays

The assays were carried out on a Carry-4000 UV-visible spectrometer (Agilent) at 30 °C using quartz cuvettes (1-, 3-, or 10-mm path-length; Hellma). All assays were carried out in 30 mm PIPES, 150 mm NaCl, pH 6.8, if not noted otherwise. For the determination of the kinetic parameters for NADH, octenoyl-CoA was kept constant at 4 mm; for octenoyl-CoA kinetics NADH was kept at 400 μm NADH and were measured at 340 nm. C2-ene adduct was measured at 385 nm, and the concentration of C2-ene adduct was determined directly within each assay using the extinction coefficient C2_385 nm_ = 7.2 cm to 1 mm^−1^ (Fig. S3*A*). All Michaelis–Menten curves were determined by curve fitting using GraphPad Prism 7.02 with a minimum of 12 measuring points. Scanning traces of InhA WT and variants using C2-ene adduct as a substrate were monitored between 300 and 500 nm using 0.17 μm InhA WT, 4.1 μm InhA Y158F, 10.8 μm InhA T196V, and 5.8 μm InhA T196A (Fig. S7).

## Author contributions

B. V., R. G. R., G. M. M. S., and T.J. E. conceptualization; B. V., R. G. R., and G. M. M. S. data curation; B. V., R. G. R., G. M. M. S., T. W., P. K., and N. S. C. formal analysis; B. V., R. G. R., and G. M. M. S. validation; B. V., R. G. R., G. M. M. S., T. W., and T. J. E. investigation; B. V., R. G. R., and G. M. M. S. visualization; B. V., R. G. R., G. M. M. S., T. W., P. K., and N. S. C. methodology; B. V. writing-original draft; R. G. R., S. S., and T. J. E. supervision; R. G. R., G. M. M. S., S. S., and T. J. E. writing-review and editing; P. K. and N. S. C. software; S. S. resources; S. S. and T. J. E. funding acquisition; T. J. E. project administration.

## Supplementary Material

Supporting Information

## Footnotes

4 Please note that the JBC is not responsible for the long-term archiving and maintenance of this site or any other third party hosted site.

## Footnotes

3 The abbreviations used are:

ACP: acyl carrier protein
PDB: Protein Data Bank
SDR: short-chain dehydrogenase
KIE: kinetic isotopic effect
ESI: electrospray ionization
MDR: medium-chain dehydrogenase.
