# Supplementary material for: InhA, the enoyl-thioester reductase from Mycobacterium tuberculosis forms a covalent adduct during catalysis
Source: J Biol Chem. 2018 Sep 14;293(44):17200–7. doi: 10.1074/jbc.RA118.005405 (PMC6222099; doi:10.1074/jbc.RA118.005405)
Supplement: Supporting Information [file supp_293_44_17200__index.html]

InhA, the enoyl-thioester reductase from Mycobacterium tuberculosis forms a covalent adduct during catalysis. — InhA forms a covalent adduct during catalysis. — InhA, the enoyl-thioester reductase from Mycobacterium tuberculosis forms a covalent adduct during catalysis — InhA forms a covalent adduct during catalysis — Supporting Information 

# InhA, the enoyl-thioester reductase from *Mycobacterium tuberculosis* forms a covalent adduct during catalysis

## Supporting Information

- Supporting Information (to be published online) - Supporting Information
