# Supplementary material for: InhA, the enoyl-thioester reductase from Mycobacterium tuberculosis forms a covalent adduct during catalysis
Source: J Biol Chem. 2018 Sep 14;293(44):17200–7. doi: 10.1074/jbc.RA118.005405 (PMC6222099; doi:10.1074/jbc.RA118.005405)
Supplement: Supporting Information [file supp_RA118.005405_140174_1_supp_198741_p77s17.docx]

**SUPPLEMENTARY INFORMATION**

**InhA, the enoyl-thioester reductase from *Mycobacterium tuberculosis* forms a covalent reaction adduct during catalysis**

Bastian Vögeli^[a]‡^,Raoul. G. Rosenthal^[a]‡^, Gabriele M. M. Stoffel^[a]‡^, Tristan Wagner^[b]^, Patrick Kiefer^[c]^, Niña Socorro Cortina^[a]^, Seigo Shima^[b]^, Tobias J. Erb*^[a]^

[a] Biochemistry and Synthetic Biology, Max-Planck-Institute for terrestrial Microbiology, Karl-von-Frisch-Strasse 10, 35043 Marburg (Germany); [b] Microbial Protein Structure, Max-Planck-Institute for terrestrial Microbiology, Karl-von-Frisch-Strasse 10, 35043 Marburg (Germany); [c] Institute of Microbiology, ETH Zürich, Vladimir-Prelog Weg 5, 8093 Zürich (Switzerland)

**Supplementary Information - Table of Contents**

**1. Supplementary Figures page 2**

**2. Supplementary Tables page 10**

**3. Supplementary References page 12**

--------------------------------------------------------------------------------------------------------------------------------------------------------------------------

**1. Supplementary Figures**

**Figure S1** Michaelis-Menten plots for InhA WT and variants. For the assays of the kinetics with octenoyl-CoA, NADH was kept constant at 300 µM, for the ones with NADH octenoyl-CoA was kept at 4 mM. All assays were measured in 30 mM PIPES, 150 mM NaCl, pH 6.8 at 30°C. Each point on the curve was measured in triplicate and the error bars represent the standard deviation. C2 adduct was added in lyophilized powder form directly out of liquid nitrogen and its starting concentration as well as its consumption was measured at 385 nm.

**Figure S2** Scheme showing the setup for the isotopic label incorporation experiment. Octenoyl-CoA is reduced by InhA WT and variants in D_2_O and a deuterium is incorporated into octanoyl-CoA in either 2R or 2S position depending on the stereospecificity of the proton doror. Octanoyl-CoA is purified via HPLC and then oxidized back to octenoyl-CoA with the stereospecific oxidase Acx4. If the proton was donated into 2R position, the resulting octenoyl-CoA will be unlabeled. In case of 2S donation the resulting octenoyl-CoA will be once deuterated.

**Figure S3** **Spectrophotometric characterization of C2-ene adduct** **A)** Absorption spectrum of C2-ene adduct. Extinction coefficients were calculated assuming the total absorbance of 33.8 cm^-1^mM-1 at 260 nm ([ε_260nm_ NADPH + ε_260nm_ CoA] = [16.9 cm-1mM-1+ 16.9 cm-1 mM-1])^1^ **B)** Uncatalyzed decay of C2-ene adduct in 30 mM PIPES buffer pH 6.8, 150 mM NaCl at 30°C was measured at 375nm, concentration was calculated using the extinction coefficient ε_375nm_= 7.46 cm^-1^mM^-1^. Data was fitted with a first order decay, the decay rate was determined to be 0.186 ± 0.001 min^-1^ with a t_1/2_ of 3.73 ± 0.01 min using three independent decay curves.

**Figure S4** **LC-MS analysis of C2-ene adduct.** The doubly charged ion with a mass-to-charge ratio of 779.17 (m/z) corresponds to a covalent adduct of NADH and octenoyl-CoA. The ion with an (m/z) at 664 corresponds to NAD+, the one at 387 corresponds to the octenoyl-pantetheine, 428 corresponds to 3´-phospho-AMP and the 542 ion corresponds to a loss of nicotinamide of the NAD+ ion.

**Figure S5 Two-dimensional NMR analysis of the C2-ene adduct** **recorded at 600 MHz in D_2_O 25 mM Na_2_DPO_4_ pH=7.9 at 4.0 °C. A)** C2-ene adduct structure and numbering **B)** ^1^H-NMR- **C)** DQF-COSY- **D)** HSQC- spectra of the C2-ene adduct**. E)** Assignment table for the C2 ene-adduct.

**
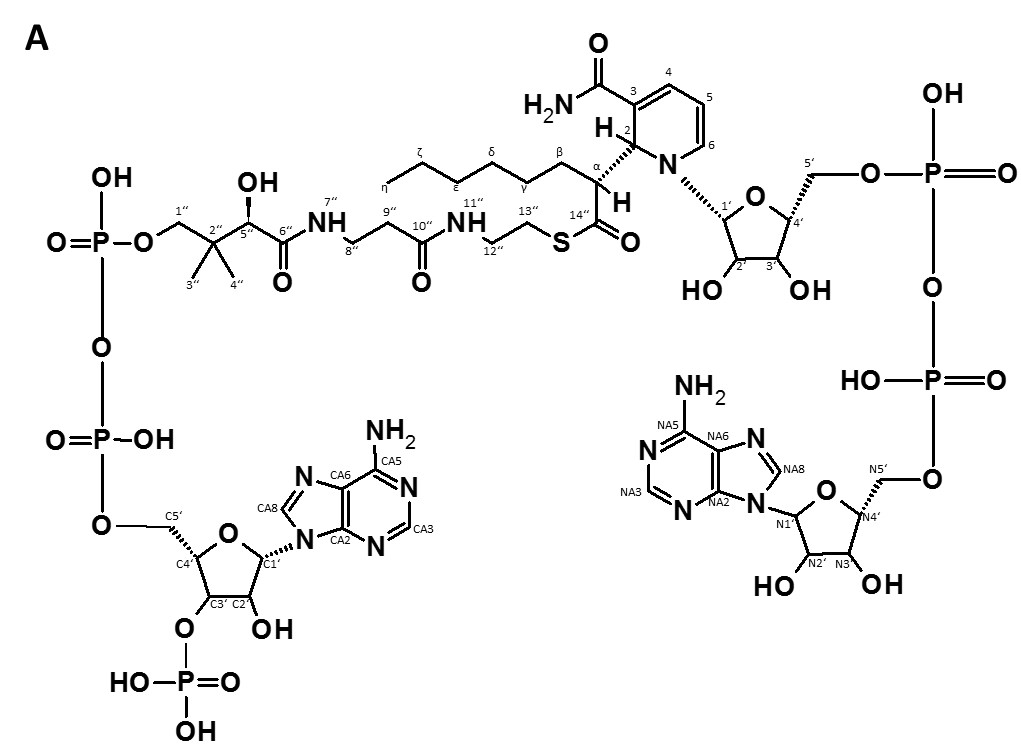
**

**
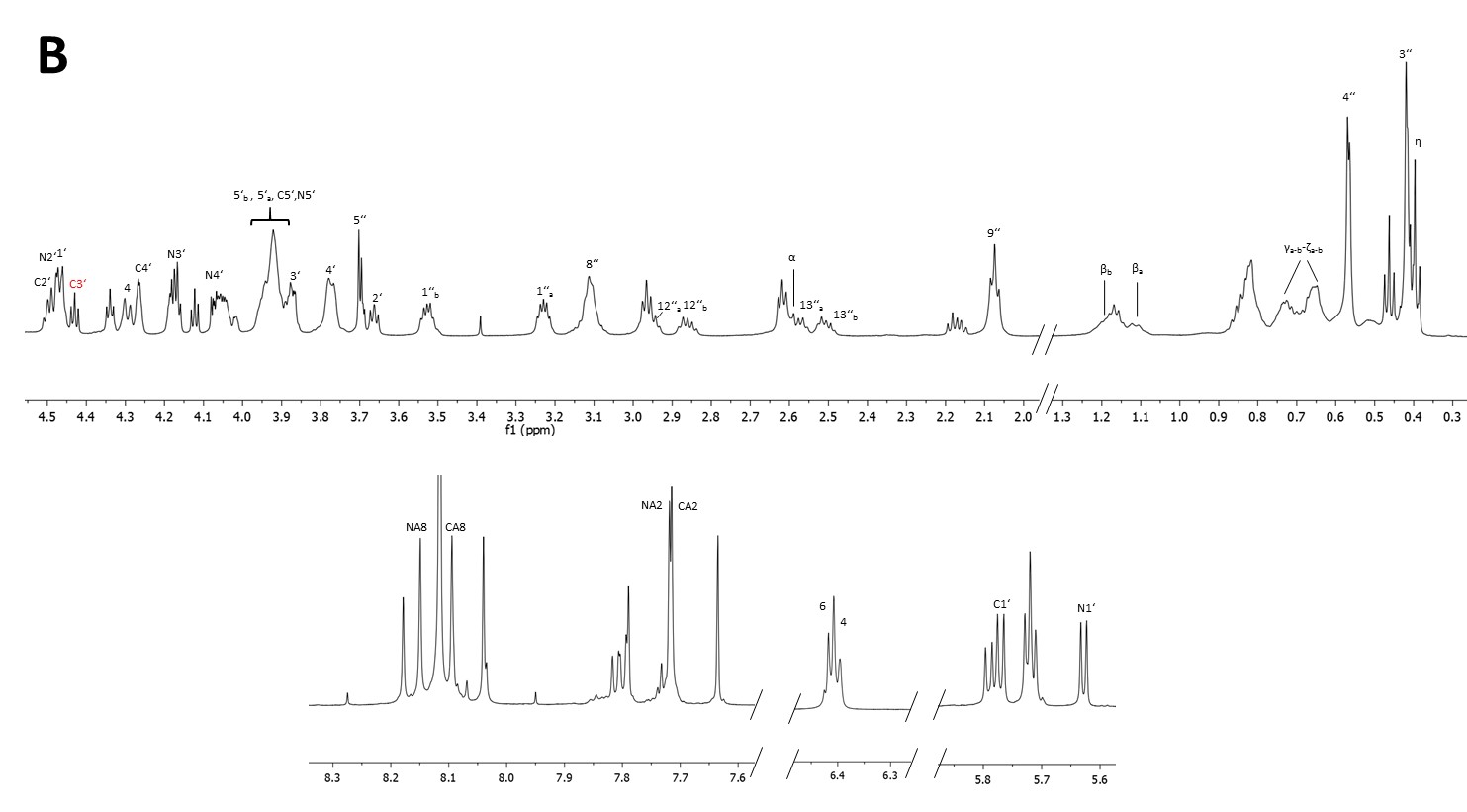
**

**
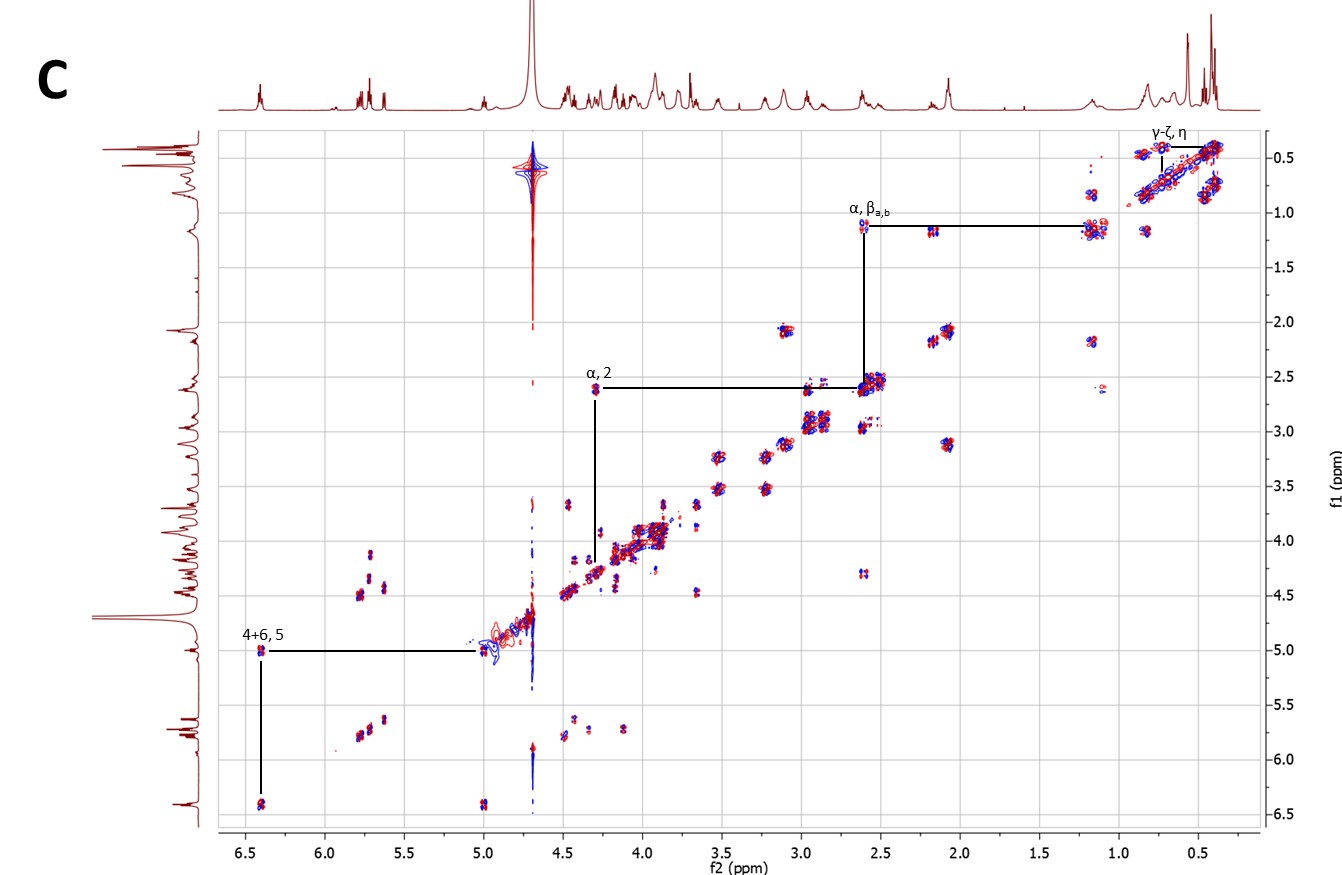
**

**
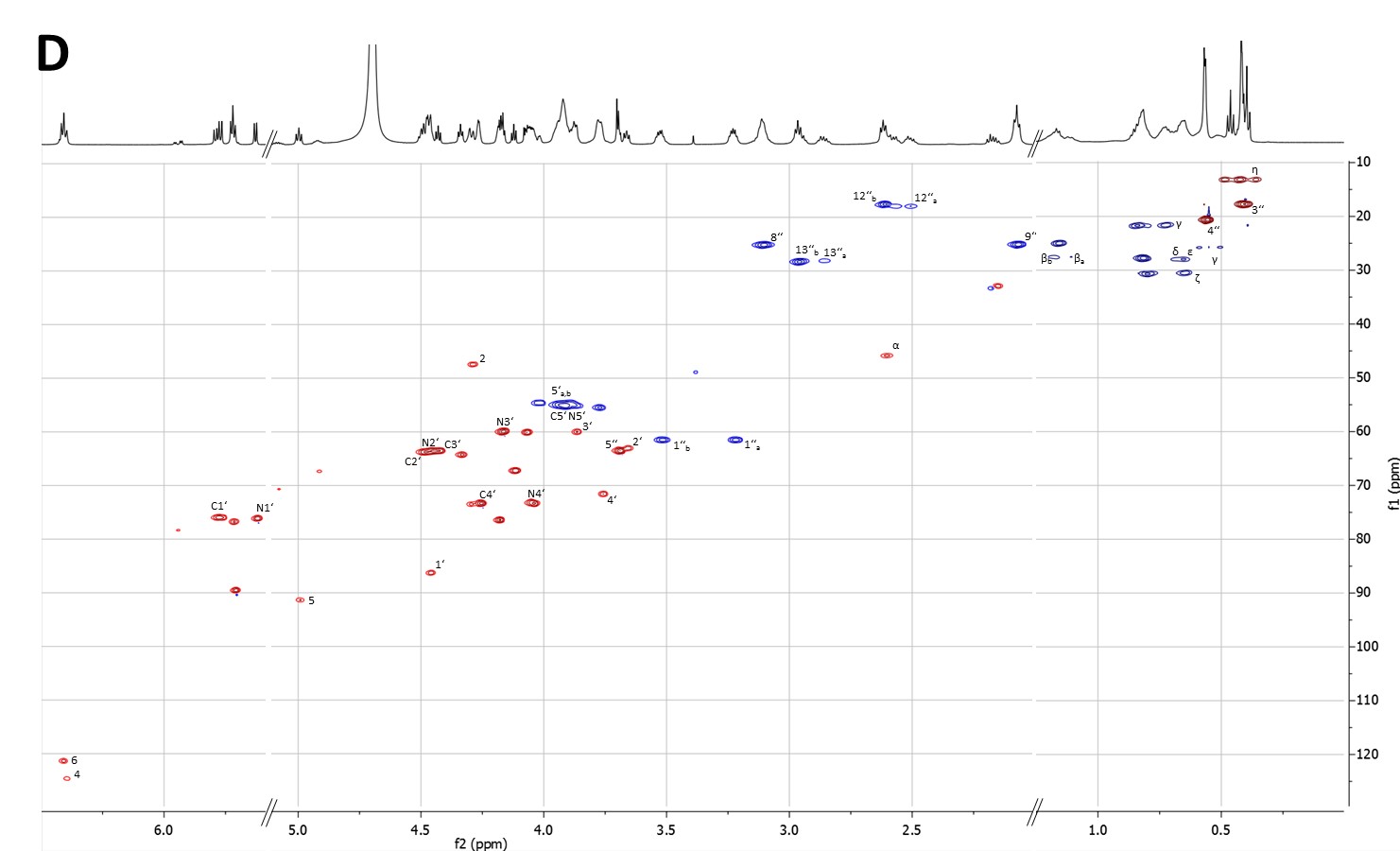
**

**E**

| **Position** | **^1^H-shift (ppm)** | **^13^C-shift (ppm)** | **HMBC** | **DQF-COSY** |
| --- | --- | --- | --- | --- |
| α | 2.62, 2.16^1^ | 56, 43^1^ | - | C_2_, β_a_, β_b_ |
| β_a_ | 1.13, 1.16^1^ | 28, 25^1^ | - | C_α_ |
| β_b_ | 1.16, 1.16^1^ | 28, 25^1^ | - | C_α_ |
| γ-ζ | 0.65-0.75, 0.80^1^ | 22,28,28,31,  22,28,28,31^1^ | - | C_η_ |
| η | 0.40, 0.46^1^ | 13, 13^1^ | - | C_γ-ζ_ |
| 2 | 4.3, 9.0^1^ | 58, 140^1^ | C_α_,C_4_,C_5_,C_6_ | C_α_ |
| 3 | - | 113^2^ | C_2_,C_5_ | - |
| 4 | 6.4 8.8^1^ | 135, 142^1^ | C_2_,C_5_,C_6_ | C_5_ |
| 5 | 5.0, 8.1^1^ | 101, 128^1^ | C_4_ | C_4_, C_6_ |
| 6 | 6.4, 8.4^1^ | 131, 146^1^ | C_2_,C_4_,C_5_ | C_5_ |
| 1’’_a_ | 3.21 | 72 | C_2’’_,C_3’’_,C_4’’_,C_5’’_ | C_1’’b_ |
| 1’’_b_ | 3.51 | 72 | C_2’’_,C_3’’_,C_4’’_,C_5’’_ | C_1’’a_ |
| 2’’ | - | 38 | C_1’’_,C_3’’_,C_4’’_,C_5’’_ | - |
| 3’’ | 0.42 | 18 | C_1’’a_,C_1’’b_,C_5’’_ | - |
| 4’’ | 0.57 | 21 | C_1’’a_,C_1’’b_,C_5’’_ | - |
| 5’’ | 3.71 | 73 | C_1’’a_,C_1’’b_,C_2’’_,C_3’’_C_4’’_, C_5’’_,C_6’’_ | - |
| 6’’ | - | 175 | C_5’’_,C_6’’_, C_8’’_ | - |
| 7’’ | - | - | - | - |
| 8’’ | 3.11 | 35 | C_9’’_,C_10’’_,C_12’’_ | C_9’’_ |
| 9’’ | 2.07 | 35 | C_8’’_,C_10’’_ | C_8’’_ |
| 10’’ | - | 174 | C_8’’_,C_9’’_,C_12’’_ | - |
| 11’’ | - | - | - | - |
| 12’’_a_ | 2.51 | 28 | C_α’’_, C_β’’_,C_γ-ζ_, C_8’’_, C_9’’_, C_10’’_ | C_12’’b_, C_13’’a_, C_13’’b_ |
| 12’’_b_ | 2.58 | 28 | C_α’’_, C_β’’_,C_γ-ζ_, C_8’’_, C_9’’_, C_10’’_ | C_12’’a_, C_13’’a_, C_13’’b_ |
| 13’’_a_ | 2.95 | 38 | C_14’’_ | C_12’’a_, C_12’’b_, C_13’’b_ |
| 13’’_b_ | 2.86 | 38 | C_14’’_ | C_12’’a_, C_12’’b_, C_13’’a_ |
| 14’’ | - | 205 | C_α_, C_β_, C_13’’a_,C_13’’b_ | - |
| 1’ | 4.46 | 96 | C_2_, C_2’_ | C_2’_ |
| 2’ | 3.66 | 73 | C_1’_ | C_1’_ |
| 3’ | 3.87 | 70 | - | C_2’_ |
| 4’ | 3.77 | 82 | - | - |
| 5’_a_ | 3.91 | 65 | C_3’_ | - |
| 5’_b_ | 3.93 | 65 | C_3’_ | - |
| C1’ | 5.77 | 86 | C_C2’_ | C_C2’_ |
| C2’ | 4.48 | 74 | C_C1’_,C_C4’_,C_C5’_ | - |
| C3’ | 4.43 | 73 | C_C4’_,C_C5’_ | - |
| C4’ | 4.26 | 83 | C_C3’_,C_C5’_ | - |
| C5’ | 3.94 | 65 | C_C3’_,C_C4’_ | - |
| N1’ | 5.65 | 86 | C_N2’_ C_N3’_ C_N4’_ | C_N2’_ |
| N2’ | 4.43 | 73 | C_N1’_ | C_N1’_,C_N3’_ |
| N3’ | 4.17 | 70 | C_N1’_ | C_N2’_ |
| N4’ | 4.04 | 83 | C_N1’_,C_N2’_,C_N5’_ | - |
| N5’ | 3.93 | 65 | C_N3’_ | - |
| CA2 | 7.72 | 152 | - | - |
| CA8 | 8.09 | 139 | - | - |
| NA2 | 7.72 | 152 | - | - |
| NA8 | 8.15 | 139 | - | - |

1 Chemical shifts in NADP+ or octanoyl-CoA

2 Chemical shift assigned from HMBC


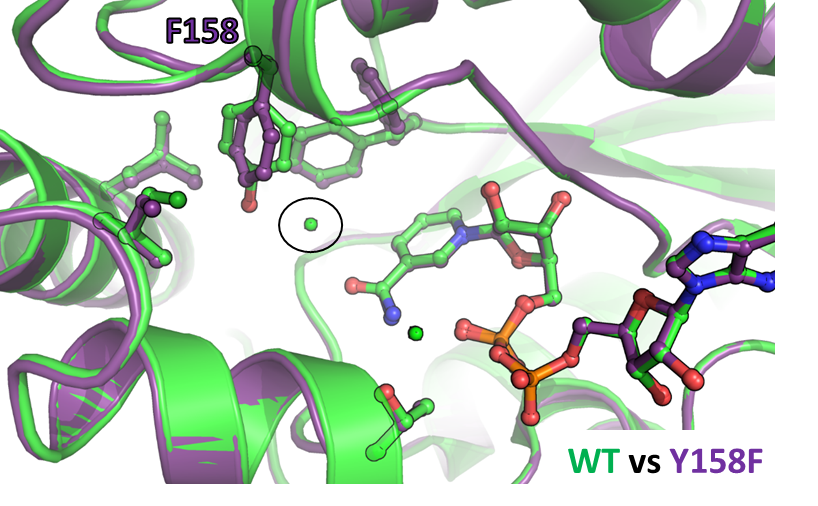


**Figure S6** Crystal structure of InhA WT (green, PDB 1BVR ^2^) and Y158F (purple, PDB 6EP8). The two active sites overlay very well with the phenylalanine in the Y158F variant slightly twisted compared to the WT tyrosine and the loss of a water molecule that is bound by the hydroxyl group of tyrosine in the phenylalanine variant (black circle)

**Figure S7** Scanning traces of InhA WT and variants on C2-ene adduct. Assays contained C2-ene adduct in 30mM PIPES pH 6.8, 150mM NaCl and was followed over time in min at 30°C. **A)** 0,17 µM InhA WT. **B)** 3,8 µM InhA Y158S **C)** 4.1µM InhA Y158F. Buildup of NADH can be observed at 340 nm. **D)** close up of the NADH formed from the backwards reaction of InhA Y158F, which is slowly consumed. **E)** 5,8 µM InhA T196A. **F)** 10.8 µM InhA T196V. Some buildup of NADH can be observed. **G)** C2-ene adduct decay without addition of any enzyme.

 **Figure S8** Possible reaction mechanism for InhA. Substrate and NADH form the C2-ene adduct via a pericyclic ene-reaction (top scheme) or via direct hydride transfer followed by a Michael addition (bottom scheme). The rehybridization (sp^2^ to sp^3^) of the Cα of the substrate causes the carbonyl to flip and expose the 2R position for protonation by tyrosine 158 upon C2-ene adduct-decay and enolate formation

**2. Supplementary Tables**

**Table S1.** Statistics of the crystal structure analysis.

|  | **InhA Y158F with NADH (6EP8)** |
| --- | --- |
| **Data collection** |  |
| Wavelength (Å) | 1.07244 |
| Space group | *P*6_2_22 |
| Resolution (Å) | 46.61 – 1.80 (1.90 – 1.80) |
| Cell dimensions |  |
| a, b, c (Å) | 98.3, 98.3, 139.8 |
| α, β, γ (°) | 90.0, 90.0, 120.0 |
| R_merge_ (%)^a^ | 6.5 (121.0) |
| R_pim_ (%)^a^ | 1.5 (27.1) |
| CC_1/2_ ^a^ | 100.0 (92.4) |
| I/σ*_I_*^a^ | 35.6 (2.9) |
| Completeness (%) ^a^ | 100.0 (100.0) |
| Redundancy^a^ | 20.5 (20.8) |
| Number of unique reflections ^a^ | 37644 (5382) |
|  |  |
| **Refinement** |  |
| Resolution (Å) | 42.55 – 1.80 |
| Number of reflections | 37562 |
| R_work_/R_free_^b^ (%) | 14.54 / 17.51 |
| Number of atoms |  |
| Protein | 2086 |
| Ligands/ions | 92 |
| Solvent | 264 |
| Mean B-value (Å^2^) | 37.1 |
| Molprobity clash score, all atoms | 2.06 (100^th^ percentile) |
| Ramachandran plot |  |
| Favored regions (%) | 255 (95.9) |
| Outlier regions (%) | 1 (0.38) |
| rmsd^c^ bond lengths (Å) | 0.009 |
| rmsd^c^ bond angles (°) | 0.129 |
| PDB code | 6EP8 |

^a^ Values relative to the highest resolution shell are within parentheses. ^b^ R_free_ was calculated as the R_work_ for 5% of the reflections that were not included in the refinement. ^c^ rmsd, root mean square deviation.

**3. Supplementary References**

1 Dawson, R. M. C. *Data for Biochemical Research*. (Clarendon Press, 1986).

2 Rozwarski, D. A., Vilcheze, C., Sugantino, M., Bittman, R. & Sacchettini, J. C. Crystal structure of the Mycobacterium tuberculosis enoyl-ACP reductase, InhA, in complex with NAD+ and a C16 fatty acyl substrate. *The Journal of biological chemistry* **274**, 15582-15589 (1999).
